# Identification of cytochrome P450 monooxygenase genes from the white-rot fungus *Phlebia brevispora*

**DOI:** 10.1186/2191-0855-2-8

**Published:** 2012-01-25

**Authors:** Ryoich Nakamura, Ryuichiro Kondo, Ming-hao Shen, Hideharu Ochiai, Shin Hisamatsu, Shigenori Sonoki

**Affiliations:** 1 Department of Environmental Sciences, School of Life and Environmental Science, Azabu University, 1-17-71 Fuchinobe, Sagamihara 252-5201, Japan; 2 Department of Forest Products Sciences, Faculty of Agriculture, Kyushu University, 6-10-1 Hakozaki, Higashi-ku, Fukuoka 812-8581, Japan; 3 College of Food Science and Engineering, Jilin Agriculture University, No.2888 Xincheng Street, Changchun, Jilin Province P.R.130118, China; 4 Research Institute of Biosciences, Azabu University, 1-17-71 Fuchinobe, Sagamihara 252-5201, Japan

**Keywords:** cytochrome P450 monooxygenase, *Phlebia brevispora*, gene cloning, real-time RT-PCR, dioxins, CYP63A

## Abstract

Three cytochrome P450 monooxygenase (CYP) genes, designated *pb*-1, *pb*-2 and *pb*-3, were isolated from the white-rot fungus, *Phlebia brevispora*, using reverse transcription PCR with degenerate primers constructed based on the consensus amino acid sequence of eukaryotic CYPs in the O_2_-binding, meander and heme-binding regions. Individual full-length CYP cDNAs were cloned and sequenced, and the relative nucleotide sequence similarity of *pb*-1 (1788 bp), *pb*-2 (1881 bp) and *pb*-3 (1791 bp) was more than 58%. Alignment of the deduced amino acid (aa) sequences of *pb*-1-*pb*-3 showed that these three CYPs belong to the same family with > 40% aa sequence similarity, and *pb*-1 and *pb*-3 are in the same subfamily, with > 55% aa sequence similarity. Furthermore, *pb*-1-*pb*-3 appeared to be a subfamily of CYP63A (CYP63A1-CYP63A4), found in *Phanerochaete chrysosporium*. The phylogenetic tree constructed by 500 bootstrap replications using the neighbor-joining method showed that the evolutionary distance between *pb*-1 and *pb*-3 was shorter than that between *pb*-2 and *pb*-1 (or *pb*-3). Exon-intron analysis of *pb*-1 and *pb*-3 showed that both genes have nearly the same number, size and order of exons and the types of introns, also indicating both genes appear to be evolutionarily close. It is interesting that the transcription level of *pb*-3 was evidently increased above the *pb*-1 transcription level by exposure to 12 coplanar PCB congeners and 2,3,7,8-tetrachlorodibenzo-*p*-dioxin, though the two genes were evolutionarily close.

## Introduction

Cytochrome P450 enzymes (CYPs) constitute a large superfamily of heme-containing monooxygenases that are widely distributed in all kingdoms of life (Nelson 2009). CYPs are involved in the metabolism of a wide variety of endogenous and xenobiotic compounds by catalyzing regio- and stereospecific monooxygenation with an oxygen atom generated from molecular oxygen. Mammalian CYPs have been studied extensively because of their leading role in drug and xenobiotic metabolism and detoxification (Allis et al. 2002; Inouye et al. 2002; McGraw JE and Waller 2006; Shimada 2006; Vrba et al. 2004; Warner et al. 2009; Yamazaki 2000; Zhang et al. 2006). CYPs from bacteria, yeast and fungi have also been well studied in the biosynthesis of essential compounds like ergosterol, which is a constituent of fungal cell membranes, and in the detoxification and biodegradation of a broad spectrum of environmental chemical pollutants (Kelly et al. 1997; Kelly et al. 2003; Lamb et al. 2000; Seth-Smith et al. 2008; van den Brink et al. 1998).

The wood-rotting *Basidiomycetes*, white-rot fungi, have been extensively used for biodegradation of various chemical pollutants. The ability to degrade such structurally diverse chemical pollutants has generally been attributed to a lignin-degrading enzyme system, including mainly lignin peroxidase, manganese-dependent peroxidase and laccase produced by these fungi (Cameron et al. 2000; Fujihiro et al. 2009; Han et al. 2004; Mayer and Staples 2002; Takagi et al. 2007; Van Aken et al. 1999). However, several studies pointed out that white-rot fungi are capable of degrading certain xenobiotics under culturing conditions that did not induce the production of lignin peroxidase, manganese-dependent peroxidase or laccase (Bumpus and Brock 1988; Mileski et al. 1988; Yadav and Reddy 1993; Yadav et al. 1995). Therefore, besides such lignin-degrading enzymes, alternative oxygenases, CYPs, are apparently involved in catalyzing degradation of several xenobiotics. In particular, several specific CYPs from *Phanerochaete chrysosporium*, the model white-rot fungus, have been studied in the metabolism of xenobiotics (Chigu et al. 2010; Kasai et al. 2010; Matsuzaki and Wariishi 2005; Ning et al. 2010; Subramanian and Yadav 2009; Syed et al. 2010). Since whole genome sequencing of *P. chrysosporium *has been completed, the molecular diversity of CYPs and the presence of at least 150 CYP genes have been elucidated (Nelson 2009).

A previous report described the fungal metabolism of coplanar PCBs (Co-PCBs) by the white-rot fungus *Phlebia brevispora *(Kamei et al. 2006). In addition, the monomethoxylated metabolite was detected in cultures containing each congener by gas chromatography and mass spectrometry, suggesting the involvement of CYP in the transformation of Co-PCBs to methoxylated compounds via hydroxylation. This result led us to search for the CYP system in *P. brevispora *involved in the metabolism of xenobiotics. Here, we describe the identification, cloning, and sequence analysis of three CYP genes from *P. brevispora*.

## Materials and methods

### Chemicals

Twelve Co-PCB congeners and 2,3,7,8-tetrachlorodibenzo-*p*-dioxin (TCDD) were purchased from Wellington Labs (Ontario, Canada). Each congener was mixed in dimethylsulfoxide (DMSO) at a concentration of 2 μg/ml for experimental use.

### Strain and culture conditions

*P. brevispora *TMIC33929 was obtained from the Tottori Mycological Institute (Tottori, Japan). The fungus was maintained as a culture on potato dextrose agar medium (Difco Laboratories, MI, USA). The fungus was grown on a potato dextrose agar plate at 26°C for 2 weeks. Then, the fungus mycelium was inoculated into Kirk liquid medium and incubated statically at 26°C for 2 to 3 weeks; an additional incubation was carried out for 2 days in Kirk liquid medium (Tien and Kirk 1988) containing all of 12 Co-PCB congeners and TCDD at a concentration of 0.25 ng/ml each. Fungal mycelium was harvested from cultures by vacuum filtration and ground in a mortar and pestle with the aid of liquid nitrogen. The ground mycelium was immediately used for RNA preparation.

### Construction of degenerate primers for cDNA isolation of CYP genes

In a previous study to search for unknown CYP genes in cultures of *P. chrysosporium*, a degenerate primer set was constructed based on the relatively conserved consensus aa sequences across eukaryotic CYPs in the O_2_-binding and heme-binding regions (Kullman and Matsumura 1997). Hence, for the first round of PCR of CYP genes, we used the same degenerate forward and a slightly modified reverse primer (see Table 1) from that used in the study of *P. chrysosporium*. For the second nested PCR of CYP genes, a degenerate forward primer was constructed based on the relatively conserved consensus aa sequence between the CYP O_2_-binding region and the CYP heme-binding region, which is called a meander region (Hasemann et al. 1995), as shown in Table 1. The degenerate reverse primer used in the second PCR was constructed for a region slightly upstream of the heme-binding region.

**Table 1:** Degenerate primers used for the searching for partial CYP cDNAs from *P. brevispora*

Target gene	Forward primer	Reverse primer
*pb-*1 (first PCR)	5'(A/C/T)TIIIIG(C/G)IGGI(A/C/G)(A/G)I(C/G)AIACIACIGC-3'	5'-CCI(A/G)(A/G/T)(A/G)CAIIII(C/T)(G/T)II(C/G/T)ICCII(A/C/G)I (C/T)(C/T)(A/G)AAIGG-3'

*pb-*1 (second PCR)	5'-T(G/T)(C/G/T)(A/C/G)(A/G)IC CI GAI(A/C)GIT(G/T)(C/G/T) (C/G/T)T-3'	5'-(C/T)(G/T)IIIICCII(A/C/G)I(C/T)(C/T)(A/G)AAIGGIA(A/G/T)I T(A/G)-3'

*pb-*2*, pb-*3	5'-(C/T)TIAA(C/T)ATI(A/C/T)TI(A/C/T)TIGCIGGI(A/C)GIGA(C/T)ACIAC-3'	5'-(C/T)TGICCIA(A/G)(A/G)CA IA(A/G/T)IC(G/T)IGGICCIGC-3'

### Isolation, cloning and sequencing of partial cDNA fragments of CYP genes

Total RNA as a template for reverse transcription (RT)-PCR was prepared from the ground mycelium using an RNeasy Plant Mini kit (QIAGEN Sciences, MD, USA). The RT mixture (13 μl), containing 1 μl total RNA (> 50 ng), 1 μl oligo(dT)_12-18 _(0.25 μg), 4 μl dNTP mixture (2.5 mM) and 7 μl sterile water, was heated at 65°C for 5 min and incubated on ice for 1 min. After addition of 4 μl 5 × first-strand buffer, 1 μl dithiothreitol (0.1 M), 1 μl RNase inhibitor and 1 μl SuperScript III reverse transcriptase (200 units) (Invitrogen Corp., CA, USA) to a total volume of 20 μl, the reaction mixture was incubated at 50°C for 60 min, then at 70°C for 15 min. Finally, 20 μl sterile water was added to the reaction mixture, which was stored at -20°C. The first PCR for CYP cDNA amplification was performed in a reaction mixture (20 μl) containing 2 μl cDNA, 1 μl each of the degenerate forward and reverse primers (10 μM), 2 μl 10 × Ex Taq buffer, 2 μl dNTP mixture (2.5 mM), 0.2 μl Ex Taq HS (TaKaRa Bio Inc., Shiga, Japan) and 11.8 μl sterile water. The cycling conditions used for the first PCR were as follows: 98°C for 3 min, followed by 30 cycles of 98°C for 30 s, 53°C for 30 s and 72°C for 120 s, with a final step at 72°C for 7 min. The second nested PCR was performed with the first PCR mixture as a template and degenerate primers for the second PCR according to the following procedure: 98°C for 3 min, followed by 30 cycles of 98°C for 30 s, 50°C for 30 s and 72°C for 120 s, with a final step at 72°C for 7 min. This two-round PCR led to the isolation of a single PCR fragment, which had high sequence homology to CYP genes from *P. chrysosporium *in BLAST homology searches. Cloning of the partial cDNA fragment for the CYP gene was performed using a Mighty TA-cloning system (TaKaRa Bio Inc.). The reaction mixture, containing 2 μl of the partial cDNA fragment, 0.5 μl pMD20-T vector and 2.5 μl ligation Mighty-Mix was incubated at 16°C for 30 min, then added to competent DH10B *E. coli *(Invitrogen Corp., CA, USA) for transformation. The transformed cells were screened in LB medium containing X-gal, IPTG and ampicillin according to the *LacZ *blue/white screening method. The cloned partial cDNA fragment was prepared from a white transformed colony grown in LB medium containing ampicillin (100 μg/ml) at 37°C overnight using a QIAprep spin miniprep kit (QIAGEN Sciences). The cloned partial cDNA fragment was sequenced according to the dye-terminator method (Sanger and Coulson 1975).

### Unknown 5'- and 3'-end sequence determination of cDNAs

The 5'- and 3'-end sequences were determined using a SMARTer RACE cDNA amplification system (Clontech Laboratories Inc., CA, USA). According to the manufacturer's instructions, 5'-RACE-ready cDNA and 3'-RACE-ready cDNA were separately prepared from total RNA (10 ng to 1 μg). The CYP cDNA-specific primers for 5'-RACE and 3'-RACE PCR were respectively designed according to the base sequence of partial cDNA as follows: 5'-RACE, 5'-TCGAGCGCGATAGTGTCGAAGTGCTGCAGC-3' (first PCR) and 5'-TGTACGCGAACTGCTGGCCGAGGCAGATG-3' (nested PCR); 3'-RACE, 5'-TCGACGAACGTCTGCACAAGCACCTGACAC-3' (first PCR) and 5'-AGCACCTGACACCGAACCCATTCATC-3' (nested PCR). The cycling conditions used for the both rounds of PCR were: 98°C for 3 min, followed by 30 cycles of 98°C for 30 s, 68°C for 30 s and 72°C for 120 s, with a final step at 72°C for 7 min. The cloning and sequencing methods were the same as described in the Materials and methods subsection: *Isolation, cloning and sequencing of partial cDNA fragments of CYP genes*.

### Cloning and sequencing of full-length cDNAs

Full-length CYP cDNAs were cloned using a universal cloning method based on the site-specific recombination system of bacteriophage lambda (Invitrogen Corp.). Based on the 5'- and 3'-end sequences, one primer set for cloning of full-length CYP cDNA was designed to the 5'-UTR region for the forward primer and to the 3'-UTR region for the reverse primer. According to the manufacturer's instructions, CYP gene specific forward and reverse primers, attached by special sequences called attB1 (5'-GGGGACAAGTTTGTACAAAAAAGCAGGCTTC-3') and attB2 (5'-GGGGACCACTTTGTACAAGAAAGCTGGGT-3') were constructed as follows: forward, 5'-GGGGACAAGTTTGTACAAAAAAGCAGGCTTCTCTCGACGGAGCCAAGTT GCCTGTATC-3'; reverse, 5'-GGGGACCACTTTGTACAAGAAAGCTGGGTTCGTCCAAATACAAGATGAAT CGCGCTAC-3'. PCR for full-length CYP cDNA was performed in a reaction mixture (50 μl) containing 1 μl cDNA, 1 μl each of the attB1-forward and attB2-reverse primers (10 μM), 25 μl PrimeSTAR Max DNA polymerase (TaKaRa Bio Inc.) and 22 μl sterile water. The cycling conditions used for PCR were: 98°C for 3 min, followed by 35 cycles of 98°C for 20 s, 61°C for 10 s and 72°C for 120 s, with a final step at 72°C for 7 min. The cloning of full-length CYP cDNA was performed using a reaction mixture containing 1-2 μl amplified PCR product (15-150 ng), 1.5 μl cloning vector (P-DONR221, 100 ng/μl), 4.5-5.5 μl TE buffer (pH 8.0) and 2 μl BP Clonase II enzyme mix (Invitrogen Corp.). The reaction mixture was incubated at 25°C for 60 min, and 1 μl proteinase K was added to stop the reaction. For transformation of *E. coli*, 1 μl of the reaction mixture was added to competent DH10B cells. The transformed cells were screened in LB medium containing kanamycin (100 μg/ml) at 37°C overnight. Full-length CYP cDNA was sequenced according to the dye-terminator sequencing method. The aa sequence was deduced by GENETYX ver.8 software (GENETYX Corp., Tokyo, Japan)

### Isolation, cloning and sequencing of full-length CYP genes from genomic DNA

The cloning and sequencing of full-length CYP genes from genomic DNA was performed using the same procedure as that described in the Materials and methods subsection: *Cloning and sequencing of full-length cDNAs *except that the cDNA was replaced with genomic DNA as the template in the reaction mixture. The genomic DNA was prepared from the ground mycelium of *P. brevispora *using a DNeasy Plant Mini kit (QIAGEN Sciences).

### Quantitative analysis of gene transcripts by real-time RT-PCR

Total RNA as a template for real-time quantitative RT-PCR was prepared from *P. brevispora *exposed to all 12 Co-PCB congeners and TCDD for 2 days at a final concentration of 0.5 ng/ml in Kirk liquid medium using an RNeasy Plant Mini kit. As a control experiment, DMSO was added into Kirk liquid medium instead of the 12 Co-PCB congeners and TCDD. Target gene-specific primers for quantification of transcripts were constructed based on < 300 bp amplicons using online technical support for design of real-time PCR assays (Roche Applied Science, Bavaria, Germany). The 18S rRNA gene was used as an internal control gene in RT-PCR. The constructed primers and amplicon lengths were: *pb-1*, 5'-CGCGTACAACGAGATGTCA-3' (forward), 5'-GAGCGCGATAGTGTCGAAGT-3' (reverse) and 64 bp (amplicon); *pb-2*, 5'-TCATCTTCGTGCCCTTCAAT-3' (forward), 5'-ACGACGCTTCGTTGTATGC-3' (reverse) and 72 bp (amplicon); *pb-3*, 5'-TTCTATGACGCGCCCTTT-3' (forward), 5'-CATGCCTATCGAACACCTCA-3' (reverse) and 65 bp (amplicon); 18S rRNA, 5'-AACTTAAAGGAATTGACGGAAGG-3' (forward), 5'-TGAGTTTCCCCGTGTTGAG-3' (reverse) and 77 bp (amplicon). The RT reaction was performed as described in the Materials and methods subsection: *Isolation, cloning and sequencing of partial cDNA fragments of CYP genes *except that oligo(dT)1_2-18 _primers were replaced with random primers in the reaction mixture. Real-time quantitative RT-PCR was performed by the detection of the nonspecific dye SYBR Green, which binds to any double-stranded DNA, using a 7500 Fast Real-Time PCR System (Applied Biosystems). The reaction mixture (25 μl), containing 2 μl cDNA, 2.5 μl each of the target gene-specific forward and reverse primers (1 μM), 12.5 μl 2 × SYBR Premix Ex Taq II (TaKaRa Bio Inc.), 0.5 μl ROX Reference Dye II and 5 μl sterile water, was put into a 96-well reaction plate, which was set in the 7500 Fast Real-Time PCR System. The cycling conditions used were: 95°C for 30 s, followed by 40 cycles of 95°C for 5 s and 60 C for 40 s. The number of gene transcripts was estimated using a λ, polyA^+^RNA (Takara Bio Inc.) as a standard reference RNA. The amplicon from a λ, polyA^+^RNA was quantified based on the SYBR green fluorescence signal. The standard curve was constructed by plotting threshold cycle values (Y-axis), which correspond to the number of PCR cycles needed to reach the threshold fluorescence, against log number of RNA molecules (X-axis). The number of gene transcripts in each of the DMSO-treated control and the12 Co-PCB, TCDD-exposed culture was individually estimated using an equation of the constructed standard curve, Y = -3.1815X + 34.935, R^2 ^= 0.99765.

## Results

### Isolation and sequence analysis of cDNAs for CYP genes *pb*-1, *pb*-2 and *pb*-3

**Figure 1:**
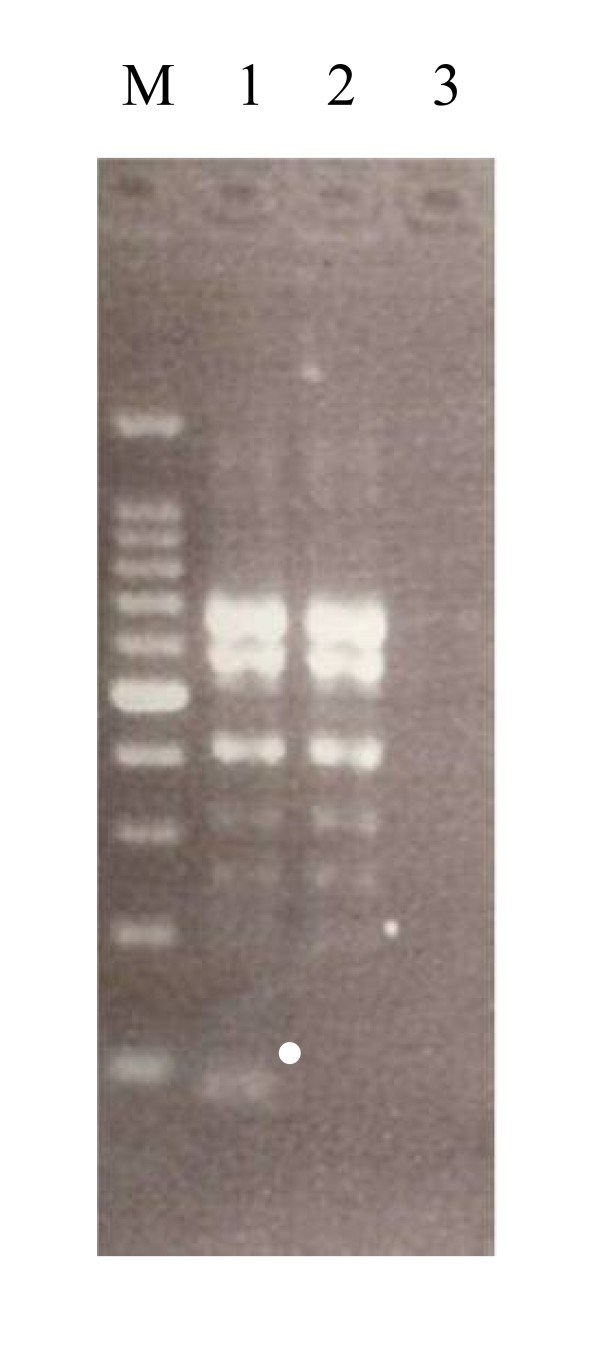
**Identification of a partial CYP cDNA from *P. brevispora *by the agarose gel electrophoresis**. PCR products were separated with 2% agarose gel for 60 min at 100 V. Each electrophoretogram shows the result of PCR with forward/reverse primers (Lane 1), a forward primer (2), a reverse primer (3), and 100 bp ladder markers (M), respectively. The partial cDNA for CYP gene, *pb*-1, having an approximate length of 100 bp is marked by a white dot.

A single cDNA fragment that had an approximate length of 100 bp was obtained by nested PCR, as shown in Figure 1. This cDNA fragment showed high nucleotide sequence homology with the CYP63 family from *P. chrysosporium *(Yadav et al. 2003). Hence, we designated this CYP gene from *P. brevispora pb*-1. Because of high nucleotide sequence homology between *pb*-1 and CYP63, degenerate primers were constructed to search for CYP genes in addition to *pb-1 *based on the highly conserved consensus sequences in the O_2_-binding region and heme-binding region of CYP63 (Yadav et al. 2003), as shown in Table 1. As a result of RT-PCR with these degenerate primers, two more CYP genes (*pb*-2, *pb*-3) were obtained. The nucleotide sequences of the 5'- and 3'-ends of the cDNA for *pb*-1, *pb*-2 and *pb*-3 were determined by a SMARTer RACE cDNA amplification system, and finally, full-length cDNAs of *pb*-1 (1788 bp), *pb*-2 (1881 bp) and *pb*-3 (1791 bp) were obtained. The nucleotide sequence similarities of these three genes were 60.9% between *pb*-1 and *pb*-2, 64.6% between *pb*-1 and *pb*-3, and 57.9% between *pb*-2 and *pb*-3. The nucleotide sequences of the three CYP cDNAs have been registered in the DNA Data Bank of Japan (DDBJ) and are available under the accession numbers AB634456, AB634457 and AB634458 for *pb*-1, *pb*-2 and *pb*-3, respectively.

### Deduced aa sequence and protein analysis

**Figure 2:**
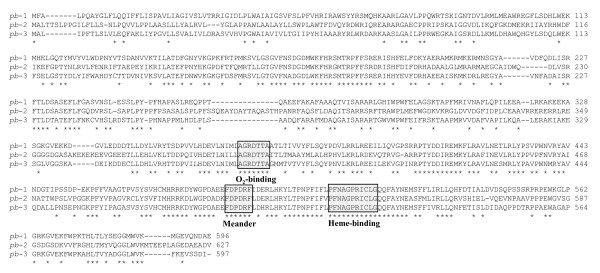
**Amino acid sequence alignments of CYP *pb-1*, *pb*-2 and *pb*-3 from *P. brevispora***. The degenerate forward and reverse primers used for the searching for partial CYP cDNAs were constructed based on the consensus motifs squared in the alignments.

**Figure 3:**
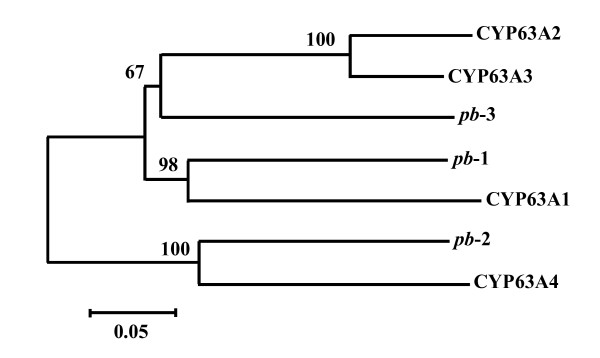
**Phylogenetic tree constructed using amino acid sequence alignments of *pb*-1-*pb*-3 and CYP63A1-CYP63A4**. The phylogenetic tree was constructed by 500 bootstrap replications and the neighbor-joining method. Amino acid sequences for CYP63A1-CYP63A4 from *P. chrysosporium *were derived from the CYP DNA data base (Nelson DR (2009) The cytochrome p450 homepage. Hum Genomics 4: 59-65).

The aa sequence similarities of *pb*-1, *pb*-2 and *pb*-3 are shown in Table 2. The percentage of aa sequence similarity was 47.4% between *pb*-1 and *pb*-2, 61.4% between *pb*-1 and *pb*-3, and 46.5% between *pb*-2 and *pb*-3. The overall aa sequence alignments showed a lower similarity in the N-terminal region (ca. < 140 aa) than in the C-terminal region. Although the aa sequence similarity was lower between *pb*-1 and *pb*-2 and between *pb*-2 and *pb*-3, the aa sequences around the meander and heme-binding regions were highly conserved in the three CYP genes (Figure 2). Furthermore, *pb*-1 and *pb*-3 also showed high aa sequence similarity to the CYP63 subfamily, CYP63A1-CYP63A3 (Doddapaneni et al. 2005; Doddapaneni and Yadav 2004), on the other hand, *pb*-2 showed high aa sequence similarity to CYP63A4 (Nelson 2009), as shown in Table 2. Phylogenetic analysis was performed for *pb*-1 through *pb*-3 and CYP63A1 through CYP63A4 using the neighbor-joining method in *MEGA *version 5 software (Tamura et al. 2011). A phylogenetic tree was constructed by 500 bootstrap replications, as shown in Figure 3. As a result, three clades appeared with high bootstrap values. CYP *pb*-1 and CYP63A1 were siblings in 98% of the bootstrap replications, and CYP *pb*-2 and CYP63A4 were siblings in 98% of the bootstrap replications. CYP *pb*-3 was grouped in a clade that included CYP63A2 and CYP63A3 in 67% of the bootstrap replications. The deduced CYP proteins for *pb*-1, *pb*-2 and *pb*-3 had estimated molecular weights of approximately 68,400, 71,300 and 68,100 and isoelectric points of 8.46, 6.56 and 6.93, respectively. The short sequences of hydrophobic aa (ca. 30 bp) at the *N*-terminal site found in all three CYP proteins are probably signal peptides for membrane binding.

**Table 2:** Amino acid sequence similarities among CYPs from *P. brevispora *and *P. chrysosporium*

	*pb*-1					
						
*pb-2*	47.4	*pb-2*				
					
*pb-3*	61.4	46.5	*pb-3*			
				
CYP63A1	60.3	46.9	64.4	CYP63A1		
			
CYP63A2	60.6	48	60.4	58.6	CYP63A2	
		
CYP63A3	63.7	49.1	61.7	59.3	84.9	CYP63A3
	
CYP63A4	48	62.4	46.9	49.6	47.5	49.7

Amino acid sequences of CYP63A1-CYP63A4 from *P. chrysosporium*used for the similarity analysis were derived from DNA data base as described in Figure 3. Each number represents the percent similarity.

### Cloning and sequence analysis of genomic CYP genes *pb*-1, *pb*-2 and *pb*-3

**Figure 4:**
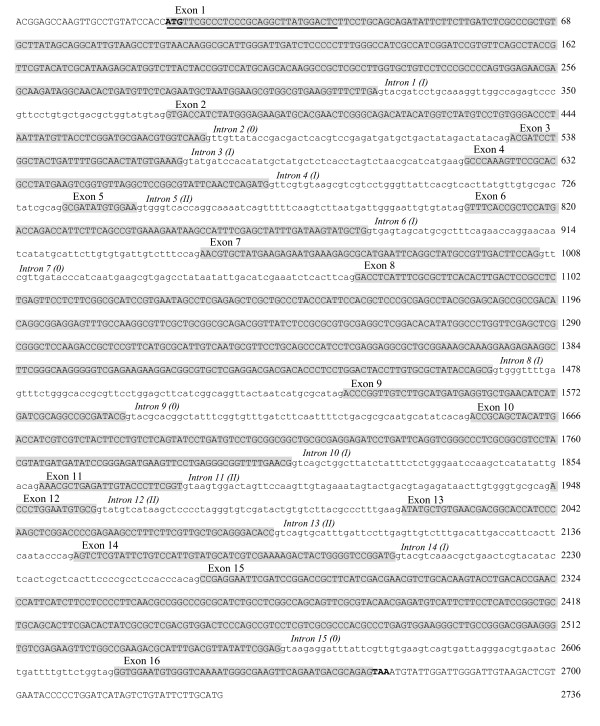
**Nucleotide sequence of CYP gene *pb*-1 from *P. brevispora***. Base numbering begins with the putative initiation codon ATG encoding the amino acid methionine. Exon and intron bases are indicated in upper and lower case letters, respectively. Exon nucleotide sequences are shadowed and numbered from 5' end. Intron nucleotide sequences are also numbered from the 5' end in *italics *followed by the intron type *(0, I or II)*. The predicted sequence for the signal peptide is underlined.

The full-length CYP gene, *pb*-1, had 16 exons and 15 introns, leading to a predicted length of 2668 bp, as shown in Figure 4. Each exon varied in size from 13 bp to 400 bp; however, the size of the 15 introns was generally around 60 bp (Table 3). The full-length CYP genes, *pb*-2 and *pb*-3, were respectively obtained using attB-sequence attached primer set prepared as follows: *pb*-2, 5'-GGGGACAAGTTTGTACAAAAAAGCAGGCTTCACATGGGGACGTCGTCAGG-3' (forward), 5'-GGGGACCACTTTGTACAAGAAAGCTGGGTTCCCACATAGATACGGCCATC-3' (reverse); *pb*-3, 5'-GGGGACAAGTTTGTACAAAAAAGCAGGCTTCTCGAAAGGCGAGCGTCTCAATTAC-3' (forward), 5'-GGGGACCACTTTGTACAAGAAAGCTGGGTCGGATTCTCCTTTGAATTTGT TCAC-3' (reverse). CYP *pb*-2 had 11 exons and 10 introns, with a length of 2871 bp, and *pb*-3 had 16 exons and 15 introns, with a length of 2595 bp. As shown in Table 3, the number, size and order of exons was the same in *pb*-1 and *pb*-3, except for three exons of 400, 72 and 45 bp in *pb*-1. Although each intron that was similar in size in *pb*-1 was slightly larger than the corresponding intron in *pb*-3, each type of intron was in the same order in *pb*-1 and *pb*-3. On the other hand, *pb*-2 was quite different from the other two CYP genes in all properties of exons and introns. The intron type was defined as follows: type 0, lies between two codons; type I, lies after the first base in the codon; type II, lies after the second base in the codon. The relative occurrence of the three intron types was 26.7% (type 0), 46.7% (type I) and 26.7% (type II) for *pb*-1 and *pb*-3, and 40% (type 0), 50% (type I) and 10% (type II) for *pb*-2.

**Table 3:** Exon-intron organization in CYP genes *pb*-I, *pb*-2 and *pb*-3

*pb-1*		*pb-2*		*pb-3*	
**Exon**	Intron (Type*)	**Exon**	**Intron (Type)**	**Exon**	**Intron (Type)**

319		328		319	
	57(I)		67(I)		57(I)
101		71		101	
	53(0)		158(0)		53(0)
37		67		37	
	49(I)		54(I)		49(I)
60		60		60	
	58(I)		62(I)		49(I)
13		149		13	
	57(II)		283(0)		55(II)
77		469		77	
	65(I)		79(I)		47(I)
59		56		59	
	62(0)		68(0)		58(0)
400		179		403	
	68(I)		85(II)		64(I)
56		146		56	
	60(0)		68(I)		54(0)
157		116		157	
	50(I)		66(0)		52(I)
25		240		25	
	64(II)				53(II)
15				15	
	53(II)				50(II)
72				75	
	59(II)				48(II)
56				56	
	60(I)				57(I)
296				296	
	65(0)				58(0)
45				42	

1788	880	1881	990	1791	804
2668		2871		2595	

Type 0	4 (26.7%)	Type 0	4(40%)	Type 0	4 (26.7%)
Type I	7 (46.7%)	Type I	5 (50%)	Type I	7 (46.7%)
Type II	4 (26.7%)	Type II	1 (10%)	Type II	4 (26.7%)

The number in the table represents the bp length each of exon and intron. *Type: representing presence of the intron between codons(Type 0), after the first base in the codon (Type I), and after the second base in the codon (Type II)

### Effect of exposure to dioxins on transcription levels of *pb*-1, *pb*-2 and *pb*-3

**Figure 5:**
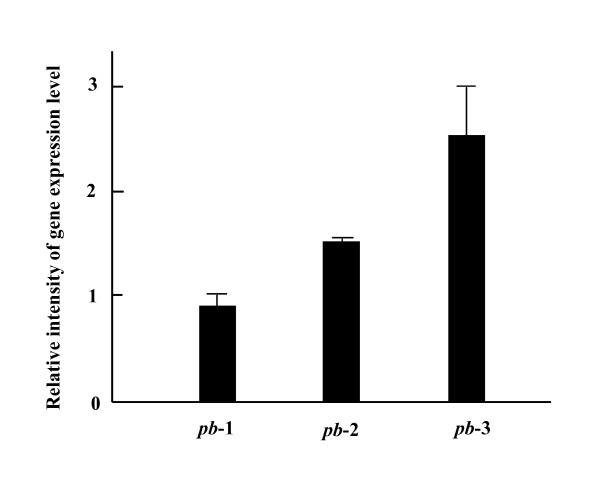
**Effect of exposure to Co-PCBs and TCDD on transcription levels of CYPs from *P. brevispora***. Each bar represents the ratio of the transcription level of CYP gene following exposure to 12 Co-PCB congeners and TCDD to that following a control exposure to DMSO. Each bar represents the mean and SD of five determinations.

The effect of exposure to 12 Co-PCB congeners and TCDD on transcription levels of *pb*-1, *pb*-2 and *pb*-3 was investigated using real-time quantitative RT-PCR to monitor the fluorescent intensity of SYBR Green. The ratio of transcription levels following exposure to 12 Co-PCB congeners and TCDD to that following a control exposure to DMSO, the solvent for the dioxins, is represented in Figure 5. Among the three CYP genes, the transcription of *pb*-3 was evidently upregulated 2- to 3-fold by exposure to the 12 Co-PCB congeners and TCDD. The transcription rate of *pb*-2 was slightly increased; however, *pb*-1 transcription was unchanged.

## Discussion

Kamei et al. (2006) reported the congener-specific metabolism of 3,3',4,4'-tetrachlorobiphenyl, 2,3,3',4,4'-pentachlorobiphenyl, 2,3',4,4',5-pentachlorobiphenyl, 3,3',4,4',5-pentachlorobiphenyl and 2,3',4,4',5,5'-hexachlorobiphenyl in 11 Co-PCBs by *P. brevispora *and the detection of methoxylated metabolites in the culture containing each congener, suggesting that these metabolites are probably produced via hydroxylation of Co-PCBs catalyzed by CYPs. To investigate the involvement of CYPs with the metabolism of dioxins, we first searched for CYP cDNA in *P. brevispora*. There is little information concerning CYP genes from *P. brevispora*; however, some useful information about nucleotide sequences of CYP cDNAs from *P. chrysosporium *is available. Hence, two sets of degenerate primers were constructed to search for CYP cDNAs from *P. brevispora*, as shown in Table 1, based on the nucleotide sequence of CYP cDNAs from *P. chrysosporium *presented by Kullman and Matsumura (1997), and the nucleotide sequences registered on the cytochrome P450 homepage organized by Nelson (2009). We describe three unique full-length cDNAs encoding CYP genes *pb*-1, *pb*-2 and *pb*-3 in *P. brevispora*. As a result of BLAST nucleotide sequence homology searching of these three CYP cDNAs, we found they were closely related to the members of the representative multigene family CYP63, CYP63A1-CYP63A4, found in *P. chrysosporium *(Doddapaneni et al. 2005; Doddapaneni and Yadav 2004; Nelson 2009). CYPs are classified and named based primarily on the level of aa sequence similarity. A family is generally defined as those CYPs having > 40% aa sequence similarity, and a subfamily is defined as those CYPs having > 55% aa sequence similarity. The deduced aa sequence alignments of *pb*-1-*pb*-3 showed that these three CYPs are members of the same family, and *pb*-1 and *pb*-3 are in the same subfamily. Furthermore, *pb*-1 and *pb*-3 appeared to belong to the subfamily of CYP63A1-CYP63A3, and *pb*-2 to CYP63A4.

Phylogenetic analysis of *pb*-1-*pb*-3 and CYP63A1-CYP63A4 with deduced aa sequence alignment using a neighbor-joining method also indicates that the phylogenetic tree is constituted of three clades and each *pb*-1-*pb*-3 belongs to a different one of the three clades. Another phylogenetic analysis using a maximum likelihood method showed a different phylogenetic tree from that of the neighbor-joining method, indicating that *pb*-3 is grouped in the clade with *pb*-1 and CYP63A1 in 81% of the bootstrap replications (data not shown). In both phylogenetic trees, the evolutionary distance between *pb*-1 and *pb*-3 was shorter than that between *pb*-2 and *pb*-1 (or *pb*-3). In addition to having a short evolutionary distance between *pb*-1 and *pb*-3, these two genes were closely located on the genomic DNA. A PCR fragment that had an approximate length of 700 bp was detected with the *pb*-3 forward primer (5'-AGGATTATGGGTCAAGTTCAAGGAAG-3') and *pb*-1 reverse primer (5'-CTTATGGACTCTTCCTGCAGCAGAT-3'), indicating that *pb*-3 is located upstream of *pb*-1 with a 613 bp interspace region in the same orientation (data not shown). Exon-intron analysis of *pb*-1 and *pb*-3 indicated that 13 of the 16 exons of the genes were similar in size and order; the exceptions were three exons: 8 (400 vs. 403 bp), 13 (72 vs. 75 bp) and 16 (45 vs. 42 bp), numbered according to the nucleotide sequence of *pb*-1 (Figure 4, Table 3). Relatively small and similarly sized introns were found in both *pb*-1 (49-68 bp) and *pb*-3 (47-64 bp), and the order of the types of introns in *pb*-1 was the same as in *pb*-3 (Table 3). From these results, the presence of some interesting variants, which were found in CYP63A1 by Yadav et al. (2003), would also be expected in the CYP genes of *P. brevispora*. In a study of intron-exon organization using the numerous *Arabidopsis *CYP genes, the intron position and type were well conserved among both subfamily and family, suggesting that intron position and type can be correlated with phylogenic relations and CYP functions among the subfamily and family (Paquette et al. 2000).

Searching for CYP genes involved in the metabolism of dioxins in *P. brevispora *is an objective of our studies; one CYP gene (*pb*-3) found in *P. brevispora *was especially upregulated at the level of transcription following exposure to 12 Co-PCB congeners and TCDD. To detect precisely the change in transcription rates of CYP genes by exposure to 12 Co-PCB congeners and TCDD, a control gene that is not influenced by these chemicals at transcription is essential for correcting the initial level of cDNA in real-time quantitative RT-PCR. The 18S rRNA gene was not influenced in the transcription step by 12 Co-PCB congeners and TCDD in preliminary experiments; hence, the 18S rRNA gene was used as an internal control gene. It is interesting that only the transcription level of *pb*-3 was evidently increased by exposure to these 12 Co-PCB congeners and TCDD, though *pb*-3 and *pb*-1 were evolutionarily close. In a previous study of xenobiotic induction of CYP63A1 and CYP63A2, some xenobiotics including PCB (Aroclor 1254), appeared to be responsible for the induction of only one gene (Doddapaneni and Yadav 2004). It seems that xenobiotic induction is not due to the phylogenetic correlation between the CYP genes, but rather due to the presence of the transcription regulatory site, e.g., xenobiotic response elements, located upstream of the CYP genes.

In this study, we have described the presence of three CYP genes in a white-rot fungus, *P. brevispora*; one of these genes was upregulated on exposure to dioxins. However, it is not obvious whether this upregulated CYP gene is involved in the metabolism of dioxins; so further experiments must be carried out to elucidate the correlation of CYP gene expression with the metabolism of dioxins.

## Competing interests

The authors declare that they have no competing interests.

## References

[B1] Allis JW Anderson BP Zhao G Ross TM Pegram RA Evidence for the involvement of CYP1A2 in the metabolism of bromodichloromethane in rat liver Toxicology 2002 176 25 37 10.1016/s0300-483x(02)00088-4 12062927

[B2] Bumpus JA Brock BJ Biodegradation of crystal violet by the white rot fungus *Phanerochaete chrysosporium* Appl Environ Microbiol 1988 54 1143 1150 10.1128/aem.54.5.1143-1150.1988 PMC202618 3389809

[B3] Cameron MD Timofeevski S Aust SD Enzymology of *Phanerochaete chrysosporium *with respect to the degradation of recalcitrant compounds and xenobiotics Appl Microbiol Biotechnol 2000 54 751 758 10.1007/s002530000459 11152065

[B4] Chigu NL Hirosue S Nakamura C Teramoto H Ichinose H Wariishi H Cytochrome P450 monooxygenases involved in anthracene metabolism by the white-rot basidiomycete *Phanerochaete chrysosporium* Appl Microbiol Biotechnol 2010 87 1907 1916 10.1007/s00253-010-2616-1 20508934

[B5] Doddapaneni H Subramanian V Yadav JS Physiological regulation, xenobiotic induction, and heterologous expression of P450 monooxygenase gene pc-3 (CYP63A3), a new member of the CYP63 gene cluster in the white-rot fungus *Phanerochaete chrysosporium* Curr Microbiol 2005 50 292 298 10.1007/s00284-005-4480-2 15968506

[B6] Doddapaneni H Yadav JS Differential regulation and xenobiotic induction of tandem P450 monooxygenase genes pc-1 (CYP63A1) and pc-2 (CYP63A2) in the white-rot fungus *Phanerochaete chrysosporium* Appl Microbiol Biotechnol 2004 65 559 565 10.1007/s00253-004-1645-z 15378295

[B7] Fujihiro S Higuchi R Hisamatsu S Sonoki S Metabolism of hydroxylated PCB congeners by cloned laccase isoforms Appl Microbiol Biotechnol 2009 82 853 860 10.1007/s00253-008-1798-2 19066882

[B8] Han MJ Choi HT Song HG Degradation of phenanthrene by *Trametes versicolor *and its laccase J Microbiol 2004 42 94 98 15357301

[B9] Hasemann CA Kurumbail RG Boddupalli SS Peterson JA Deisenhofer J Structure and function of cytochromes P450: a comparative analysis of three crystal structures Structure 1995 3 41 62 10.1016/s0969-2126(01)00134-4 7743131

[B10] Inouye K Shinkyo R Takita T Ohta M Sakaki T Metabolism of polychlorinated dibenzo-p-dioxins (PCDDs) by human cytochrome P450-dependent monooxygenase systems J Agric Food Chem 2002 50 5496 5502 10.1021/jf020415z 12207498

[B11] Kamei I Sonoki S Haraguchi K Kondo R Fungal bioconversion of toxic polychlorinated biphenyls by white-rot fungus, *Phlebia brevispora* Appl Microbiol Biotechnol 2006 73 932 940 10.1007/s00253-006-0529-9 16862425

[B12] Kasai N Ikushiro S Shinkyo R Yasuda K Hirosue S Arisawa A Ichinose H Wariishi H Sakaki T Metabolism of mono- and dichloro-dibenzo-p-dioxins by *Phanerochaete chrysosporium *cytochromes P450 Appl Microbiol Biotechnol 2010 86 773 780 10.1007/s00253-009-2413-x 20201136

[B13] Kelly SL Lamb DC Jackson CJ Warrilow AG Kelly DE The biodiversity of microbial cytochromes P450 Adv Microb Physiol 2003 47 131 186 10.1016/s0065-2911(03)47003-3 14560664

[B14] Kelly SL Lamb DC Kelly DE Sterol 22-desaturase, cytochrome P45061, possesses activity in xenobiotic metabolism FEBS Lett 1997 412 233 235 10.1016/s0014-5793(97)00785-0 9257726

[B15] Kullman SW Matsumura F Identification of a novel cytochrome P-450 gene from the white rot fungus *Phanerochaete chrysosporium* Appl Environ Microbiol 1997 63 2741 2746 10.1128/aem.63.7.2741-2746.1997 PMC168569 9212420

[B16] Lamb DC Kelly DE Masaphy S Jones GL Kelly SL Engineering of heterologous cytochrome P450 in Acinetobacter sp.: application for pollutant degradation Biochem Biophys Res Commun 2000 276 797 802 10.1006/bbrc.2000.3541 11027550

[B17] Matsuzaki F Wariishi H Molecular characterization of cytochrome P450 catalyzing hydroxylation of benzoates from the white-rot fungus *Phanerochaete chrysosporium* Biochem Biophys Res Commun 2005 334 1184 1190 10.1016/j.bbrc.2005.07.013 16039998

[B18] Mayer AM Staples RC Laccase: new functions for an old enzyme Phytochemistry 2002 60 551 565 10.1016/s0031-9422(02)00171-1 12126701

[B19] McGraw JES Waller DP Specific human CYP 450 isoform metabolism of a pentachlorobiphenyl (PCB-IUPAC# 101) Biochem Biophys Res Commun 2006 344 129 133 10.1016/j.bbrc.2006.03.122 16616008

[B20] Mileski GJ Bumpus JA Jurek MA Aust SD Biodegradation of pentachlorophenol by the white rot fungus *Phanerochaete chrysosporium* Appl Environ Microbiol 1988 54 2885 2889 10.1128/aem.54.12.2885-2889.1988 PMC204399 3223759

[B21] Nelson DR The cytochrome p450 homepage Hum Genomics 2009 4 59 65 10.1186/1479-7364-4-1-59 PMC3500189 19951895

[B22] Ning D Wang H Zhuang Y Induction of functional cytochrome P450 and its involvement in degradation of benzoic acid by *Phanerochaete chrysosporium* Biodegradation 2010 21 297 308 10.1007/s10532-009-9301-z 19787435

[B23] Paquette SM Bak S Feyereisen R Intron-exon organization and phylogeny in a large superfamily, the paralogous cytochrome P450 genes of *Arabidopsis thaliana* DNA Cell Biol 2000 19 307 317 10.1089/10445490050021221 10855798

[B24] Sanger F Coulson AR A rapid method for determining sequences in DNA by primed synthesis with DNA polymerase J Mol Biol 1975 94 441 448 10.1016/0022-2836(75)90213-2 1100841

[B25] Seth-Smith HM Edwards J Rosser SJ Rathbone DA Bruce NC The explosive-degrading cytochrome P450 system is highly conserved among strains of Rhodococcus spp Appl Environ Microbiol 2008 74 4550 4552 10.1128/AEM.00391-08 PMC2493155 18487400

[B26] Shimada T Xenobiotic-metabolizing enzymes involved in activation and detoxification of carcinogenic polycyclic aromatic hydrocarbons Drug Metab Pharmacokinet 2006 21 257 276 10.2133/dmpk.21.257 16946553

[B27] Subramanian V Yadav JS Role of P450 monooxygenases in the degradation of the endocrine-disrupting chemical nonylphenol by the white rot fungus *Phanerochaete chrysosporium* Appl Environ Microbiol 2009 75 5570 5580 10.1128/AEM.02942-08 PMC2737932 19542331

[B28] Syed K Doddapaneni H Subramanian V Lam YW Yadav JS Genome-to-function characterization of novel fungal P450 monooxygenases oxidizing polycyclic aromatic hydrocarbons (PAHs) Biochem Biophys Res Commun 2010 399 492 497 10.1016/j.bbrc.2010.07.094 PMC2943217 20674550

[B29] Takagi S Shirota C Sakaguchi K Suzuki J Sue T Nagasaka H Hisamatsu S Sonoki S Exoenzymes of *Trametes versicolor *can metabolize coplanar PCB congeners and hydroxy PCB Chemosphere 2007 67 S54 57 10.1016/j.chemosphere.2006.05.090 17250871

[B30] Tamura K Peterson D Peterson N Stecher G Nei M Kumar S MEGA5: Molecular evolutionary genetics analysis using maximum likelihood, evolutionary distance, and maximum parsimony methods Mol Biol Evol 2011 28 2731 2739 10.1093/molbev/msr121 PMC3203626 21546353

[B31] Tien M Kirk TK Lignin peroxidase of *Phanerochaete chrysosporium* Methods in Enzymology 1988 161 238 249

[B32] Van Aken B Hofrichter M Scheibner K Hatakka AI Naveau H Agathos SN Transformation and mineralization of 2,4,6-trinitrotoluene (TNT) by manganese peroxidase from the white-rot basidiomycete *Phlebia radiata* Biodegradation 1999 10 83 91 10.1023/a:1008371209913 10466197

[B33] van den Brink HM van Gorcom RF van den Hondel CA Punt PJ Cytochrome P450 enzyme systems in fungi Fungal Genet Biol 1998 23 1 17 10.1006/fgbi.1997.1021 9501474

[B34] Vrba J Kosina P Ulrichova J Modriansky M Involvement of cytochrome P450 1A in sanguinarine detoxication Toxicol Lett 2004 151 375 387 10.1016/j.toxlet.2004.03.005 15183462

[B35] Warner NA Martin JW Wong CS Chiral polychlorinated biphenyls are biotransformed enantioselectively by mammalian cytochrome P-450 isozymes to form hydroxylated metabolites Environ Sci Technol 2009 43 114 121 10.1021/es802237u 19209593

[B36] Yadav JS Quensen JF Tiedje JM Reddy CA Degradation of polychlorinated biphenyl mixtures (Aroclors 1242, 1254, and 1260) by the white rot fungus *Phanerochaete chrysosporium *as evidenced by congener-specific analysis Appl Environ Microbiol 1995 61 2560 2565 10.1128/aem.61.7.2560-2565.1995 PMC167527 7618867

[B37] Yadav JS Reddy CA Mineralization of 2,4-Dichlorophenoxyacetic Acid (2,4-D) and Mixtures of 2,4-D and 2,4,5-Trichlorophenoxyacetic Acid by *Phanerochaete chrysosporium* Appl Environ Microbiol 1993 59 2904 2908 10.1128/aem.59.9.2904-2908.1993 PMC182384 16349039

[B38] Yadav JS Soellner MB Loper JC Mishra PK Tandem cytochrome P450 monooxygenase genes and splice variants in the white rot fungus *Phanerochaete chrysosporium*: cloning, sequence analysis, and regulation of differential expression Fungal Genet Biol 2003 38 10 21 10.1016/s1087-1845(02)00508-x 12553932

[B39] Yamazaki H Roles of human cytochrome P450 enzymes involved in drug metabolism and toxicological studies Yakugaku Zasshi 2000 120 1347 1357 10.1248/yakushi1947.120.12_1347 11193384

[B40] Zhang JY Wang Y Prakash C Xenobiotic-metabolizing enzymes in human lung Curr Drug Metab 2006 7 939 948 10.2174/138920006779010575 17168693

